# Glycoproteomics analysis of complement factor H and its complement-regulatory function during *Streptococcus pneumoniae*-associated hemolytic uremic syndrome

**DOI:** 10.3389/fimmu.2025.1645196

**Published:** 2025-08-21

**Authors:** Laura M. Baas, Kioa L. Wijnsma, Fokje Zijlstra, Nicole C. A. J. van de Kar, Lieke ter Steeg, Antonia H. M. Bouts, Marloes A. H. M. Michels, Jeroen D. Langereis, Dirk Lefeber, Hans J. C. T. Wessels, Lambertus P. van den Heuvel

**Affiliations:** ^1^ Department of Pediatric Nephrology, Radboud University Medical Centre, Amalia Children’s Hospital, Nijmegen, Netherlands; ^2^ Department of Human Genetics, Radboud University Medical Centre, Nijmegen, Netherlands; ^3^ Department of Pediatric Nephrology, Emma Children’s Hospital, Amsterdam University Medical Centre, Amsterdam Institute of Infection and Immunity, Amsterdam, Netherlands; ^4^ Laboratory of Medical Immunology, Department of Laboratory Medicine, Radboudumc Community for Infectious Diseases, Radboud University Medical Centre, Nijmegen, Netherlands; ^5^ Department of Neurology, Donders Institute for Brain, Cognition and Behaviour, Radboud University Medical Centre, Nijmegen, Netherlands; ^6^ Department of Pediatrics/Pediatric Nephrology, University Hospital Leuven, Leuven, Belgium; ^7^ Department of Development and Regeneration, University Hospital Leuven, Leuven, Belgium

**Keywords:** complement factor H, *Streptococcus pneumoniae*, hemolytic uremic syndrome, glycosylation, glycoproteomics, functional assays

## Abstract

Hemolytic uremic syndrome caused by an invasive *Streptococcus pneumoniae* infection (SP-HUS) is a rare and severe disease that primarily affects children under two years of age. The pathophysiology of SP-HUS remains poorly understood, and treatment is largely supportive. Complement factor H (FH) is a key regulator of the alternative pathway of the complement system. It has been hypothesized that loss of sialic acids from FH’s N-glycans may impair its regulatory functions, thereby potentially leading to complement-mediated endothelial cell damage in SP-HUS. In this study, we investigated the N-glycosylation patterns of FH across three N-glycosylation sites for four SP-HUS patients and compared it to healthy controls using LC-MS/MS-based glycopeptide profiling. We identified significant changes in FH glycosylation during the acute phase of SP-HUS, including an increased presence of N-glycans lacking sialic acids, galactose and N-acetylglucosamine (GlcNAc) relative to the controls. This abnormal glycosylation was most prominent during the acute phase in all patients and showed partial or complete normalization during remission. Interestingly, despite these major glycosylation changes, functional assays revealed no significant impairment in the complement regulatory activity of FH, as measured by its ability to facilitate C3b degradation and to prevent complement-mediated hemolysis of sheep erythrocytes. In conclusion, our findings show that FH’s N-glycosylation is severely altered in the acute phase in SP-HUS patients, comprising more than just the loss of sialic acids. However, these changes do not directly affect FH’s complement regulatory function. These results highlight the complex yet poorly understood role of N-glycosylation during infection, and the contribution of FH’s N-glycans to complement (dys)regulation and disease pathogenesis.

## 1 Introduction

Hemolytic uremic syndrome (HUS) is a disease that belongs to the group of thrombotic microangiopathies (TMA) and is characterized by the presence of a triad of symptoms, comprising mechanical hemolytic anemia, thrombocytopenia, and acute kidney injury (AKI) (1, 2). HUS can have several causes, including infection with Shiga toxin-producing *E. coli* (STEC-HUS), dysregulation of the complement system (complement-mediated atypical HUS; CaHUS) or invasive *Streptococcus pneumoniae* infection (SP-HUS) (3). SP-HUS is an acute and severe disease that has a high mortality and morbidity rate, with the highest prevalence among children younger than two years of age (4–7). For SP-HUS only supportive treatment is available.

Due to the rarity of the disease, with an estimated incidence of 0.015-0.065 cases per 100,000 children, its pathophysiology remains poorly defined (4, 5). While there are many theories surrounding the pathogenesis, one of the main hypothesized contributors to the development of SP-HUS is exogenic neuraminidase. Pneumococcal neuraminidases (NanA, NanB and/or NanC) are released by the bacterium during infection and can cleave N-acetylneuraminic acids (Neu5Ac or sialic acids) from glycans located on proteins and/or cellular surfaces. Cleavage of sialic acids from surface glycans, located on e.g. erythrocytes and the glomerular endothelium (8, 9), leads to the exposure of the Thomsen Friedenreich (TF)-antigen, to which preformed anti-TF IgM antibodies can bind. These antibodies can subsequently cause activation of the complement system through the classical pathway (8, 10). Desialylation of cellular surfaces may also cause dysregulation of the alternative pathway (AP), because the main regulator of the AP, complement factor H (FH), binds to sialic acids on cell surfaces to protect them from complement attack (8, 10, 11).

FH is a 150 kDa plasma glycoprotein that is essential for controlling the AP in blood and on cellular surfaces. FH inhibits complement activation by acting as a cofactor for complement factor I (FI) in the cleavage and inactivation of C3b, and by preventing the formation and accelerating the decay of C3 convertases. FH is especially important for regulating complement activation on host cell surfaces and discrimination of self and non-self through recognition of sialic acids and sulfated glycosaminoglycans (12–14). The loss of sialic acids on host cells such as endothelial cells is therefore thought to lead to complement-mediated endothelial cell damage (7, 15).

Besides these potential effects of sialic acid loss on host cells, recent findings have also shown a possible role for sialic acid loss of the N-glycans on FH itself in SP-HUS (16). FH is a heavily glycosylated glycoprotein that contains eight N-glycosylation sites predominantly occupied by di-antennary di-sialylated complex N-glycans (17). *In vitro* desialylated FH exhibited a decreased capability to control complement activation on sheep erythrocyte surfaces, suggesting a role for the sialic acids on FH in binding to cellular surfaces. However, contradictory reports have been published as well. Studies have reported that FH with truncated or desialylated N-glycans, and FH that is completely deglycosylated or contains only the core GlcNAc residue, retains normal complement regulatory activity on sheep erythrocyte surfaces (14, 18, 19). Thus, the function of FH’s N-glycans in complement regulation remains unclear.

To further investigate the changes in FH glycosylation during SP-HUS, we evaluated the glycosylation status of FH in four SP-HUS patients during the acute phase and remission using LC-MS/MS glycopeptide profiling and compared it to healthy controls. Subsequently, we immunoprecipitated FH from SP-HUS sera for functional analysis and compared it with FH isolated from healthy control serum and serum treated *in vitro* with neuraminidase. We evaluated FH’s ability to regulate complement activation on the surface of erythrocytes via a hemolytic assay and FH’s ability to exert cofactor activity for FI by measuring the degradation of C3b to iC3b. In addition, we replicated a previously published method for the desialylation of purified FH, repeated our functional assays, and compared the results to obtain mechanistic insights in the differences in outcome.

## 2 Methods

### 2.1 Ethics

Passive (opt-out) informed consent for data collection in the TMA database was obtained from three SP-HUS patients previously treated at Radboud University Medical Center, Nijmegen, the Netherlands. Separate informed consent was obtained from one SP-HUS patient who had been treated at Emma Children’s Hospital, Amsterdam, the Netherlands. The TMA database was approved by the Medical Research Ethics Committee of Oost-Nederland (2019-5676). SP-HUS patient sera were collected retrospectively from the routine diagnostic laboratory, and written informed consent was obtained from 14 healthy controls for blood withdrawal and usage of the samples for research purposes with approval of the protocol by the ethical committee of the Radboud University Medical Centre (FH 06979). This study was performed in accordance with the appropriate version of the declaration of Helsinki (20).

### 2.2 SP-HUS patients

Four patients diagnosed with SP-HUS were included in this study based on the availability of stored sera. Median (range) age at onset was 20 (6-39) months. All patients presented with pneumoniae, with empyema occurring in two, and developed hematological TMA and AKI during the course of disease. An invasive *S. pneumoniae* infection was confirmed in three patients with a urine antigen test (P1 and P2) or blood culture (P4), while in P3 it was suspected but not proven. Fecal diagnostics and/or serology did not indicate a STEC infection in any of the patients. Genetic screening of CaHUS-associated genes (variants in *C3*, *CFH*, *MCP*, *CFI*, *CFB*, *CFH-related proteins 1-5*, and *diacylglycerol kinase-ϵ*, and genomic rearrangements in the *CFH/CFH-related* gene cluster) was performed in all patients except P3. P1, P2 and P4 showed no disease-causing genetic variants in the genetic screening (see **Supplementary Table 1** for clinical data).

Acute phase samples were collected within one week after TMA presentation, during which TMA and AKI were still present. All patients received antibiotics before collection of acute phase samples, and P2 and P4 received one or more erythrocyte transfusions beforehand. None began dialysis prior to sample collection. Remission samples were collected at a median (range) of 68 (15-138) days after TMA presentation and only after platelet count had normalized (see **Supplementary Table 2** for sample collection times). TMA was defined by at least two of the following criteria: thrombocytopenia (platelet count <150 ×10^9^/L), lactate dehydrogenase exceeding the upper limit of normal (>250 U/l), and low/undetectable haptoglobin (<0.3 mg/l). AKI was defined as a rise in serum creatinine of ≥26.53 µmol/l (0.3 mg/dl) within 48 hours or ≥1.5 times baseline in <7 days.

FH levels were determined during both the acute phase and remission for all patients from EDTA plasma using ELISA as described previously (21). In the acute phase, P1 exhibited moderately decreased FH levels, whereas P2, P3, and P4 demonstrated normal FH levels. During remission, FH levels increased when compared to the acute phase sample, with normal FH levels for all patients (**Supplementary Figure 1**).

### 2.3 Sample preparation

Blood samples from SP-HUS patients and healthy controls were prepared as described previously and stored at -80°C until further use (22). Blood samples from 14 healthy controls were pooled and aliquoted after collection (normal human serum, NHS). Neuraminidase-treated NHS (Neu-NHS) was prepared by incubating NHS 1:1 with neuraminidase from *C. welchii* (Sigma, 1U/mL in 0.1 M Tris-HCl pH 7,4) at 37°C for 24 hours. In a parallel sample, neuraminidase was omitted to serve as a control. Neu-NHS was generated to obtain and functionally evaluate *in vitro* desialylated FH (dFH). All samples were stored at -80°C until further use.

In addition, we prepared *in vitro* desialylated FH according to the protocol of Gómez Delgado et al., in which neuraminidase is added to serum-purified FH directly (16). In short, 25 µg serum-purified FH (CompTech) was added to 25 µL *C. welchii* neuraminidase (Sigma, 1U/mL in 0.1 M Tris-HCl pH 7.4), 100 µL 0.1 M sodium acetate, pH 5.0 and 25 µL 1% bovine serum albumin (BSA; Merck) and incubated for 4 hours at 37°C with gentle shaking. In parallel, a control sample without the addition of neuraminidase was included. The reaction was stopped by the addition of 25 µL 0.5 M sodium hydrogen carbonate, pH 9.8. The dFH sample was stored at -80°C until further use.

### 2.4 Glycopeptide analysis

Samples were prepared for glycoproteomics experiments as described previously (23). Briefly, human plasma proteins were denatured in 8 M urea 10 mM Tris-HCl pH 8 and cysteine residues were reduced and alkylated using dithiothreitol and 2-chloroacetamide, respectively. After 3-fold dilution of the sample in 50 mM ammonium bicarbonate buffer, 1 µg trypsin per 50 µg plasma protein was added and samples were incubated overnight at 37°C. Glycopeptides were enriched using 100 µl Sepharose CL-4B beads (Merck) in 0.20 µm pore size 96 multiwell filter plates (AcroPrep Advance, VWR). After the tryptic digest was applied, beads were washed subsequently 3 times with 83% acetonitrile (ACN) and 83% ACN with 0.1% trifluoroacetic acid. Glycopeptides were eluted with 50 µl of water.

Glycopeptide analyses were performed as described previously (24) using a nanoElute liquid chromatography system (Bruker Daltonics) connected online to a timsTOF Pro2 instrument (Bruker Daltonics) *via* a nanoflow electrospray ionization source with ACN enriched nitrogen gas from the nanoBooster. Peptides were separated on a 75 μm × 150 mm C18 reversed-phase column (1.9 μm, 120 Å pore size; Bruker Daltonics) at 45°C. The gradient elution ranged from 7% to 45% acetonitrile in 0.1% formic acid and 0.02% TFA over 25 minutes at a flow rate of 500 nL/min. Measurements were performed as described previously in positive ionization mode using data dependent acquisition – Parallel Accumulation SErial Fragmentation (dda-PASEF) with parameters optimized for glycopeptides as described by Baerenfaenger et al. (24). Settings included a TIMS range of 0.7–1.5 1/K0, mass analyzer range of 50–4000 *m/z*, accumulation and ramp time of 100 ms, ion charge control at 40 million, dynamic exclusion set to 24 s, TIMS stepping disabled, 7.5ms PASEF measurement time, 50000 target PASEF intensity, 30-90eV collision energy from 0.6-1.6 1/K0, 1700 Vpp collision RF, 80 µs transfer time, 10 µs pre-pulse storage time. Glycopeptides were identified using MSFragger Glyco (FragPipe v15.0, MSFragger v3.4, Philosopher v4.1.1) (25). Searches were performed with semitryptic specificity and a precursor mass tolerance of 20 ppm, allowing isotope errors from 0–2, peptide length 5–50 amino acids, and 600–20000 m/z range. Modifications included fixed carbamidomethylation and variable methionine oxidation and N-terminal ammonia loss. A database of 4,029 human secreted proteins (UniProt (26), November 2021) was used in combination with 475 unique human N-glycan masses from the GlyGen database (April 2022) (27). Peptide-spectrum matches (PSMs), glycans, and peptide sequences were filtered to 1% FDR. Glycopeptide identification from FH were manually verified and subsequently used to create a precursor targetlist for targeted label-free quantitation (LFQ) using in-house developed DataAnalysis (Bruker Daltonics) scripts (28). Glycoforms are expressed as relative LFQ fractions (%) for each respective peptide (IPCQPPQIEHGTINSRR, ISEENETTCYMGK, and MDGASNVTCINSR).

### 2.5 Immunoprecipitation of factor H

Protein A/G beads (70 µL, Pierce) were bound with 30 µg anti-FH antibody (OX-24, Bio-Rad) by incubation in phosphate buffered saline (PBS), pH 7.4 for 3 hours at room temperature (RT). After incubation, the beads were washed three times with 0.2 M sodium borate pH 9.0 and the supernatant was discarded. The bound antibodies were subsequently crosslinked to the protein A/G beads by incubating the antibody-bead complexes with 0.2 M sodium borate containing 20 mM dimethyl pimelimitade dihydrochloride (Sigma Aldrich) for 40 minutes on a rocking platform at RT. Afterwards, the beads were washed once with 0.2 M ethanolamine, pH 8.0, and incubated for 1 hour at RT in 0.2 M ethanolamine, pH 8.0. To remove uncoupled immunoglobulins, the beads were washed three times with wash buffer (0.58% (v/v) acetic acid, 150 mM NaCl) and three times with cold PBS. The OX-24-coupled beads were stored in PBS pH 7.4 at 4°C until further use.

For immunoprecipitation of FH, 12.5% (v/v) patient serum, Neu-NHS or NHS was added to RIPA buffer (10 mM Tris pH 8, 140 mM NaCl, 1 mM EDTA, 0.5 mM EGTA, 1% (v/v) Triton X-100, 0.1% (w/v) SDS, 0.1% (w/v) Sodium deoxycholate, cOmplete mini protease inhibitors (Roche)). To reduce non-specific binding to the beads, the immunoprecipitation samples were pre-cleared by incubation with 20 µL protein A/G beads for 30 minutes at 4°C, under gentle rotation. After removal of the beads, the beads previously crosslinked to anti-FH antibodies were added to the sample and incubated overnight at 4°C on a rotating platform. The supernatant (unbound fraction) was removed from the beads and kept on ice, while the beads were washed three times with low salt wash buffer (1% (v/v) triton X-100, 0.1% (w/v) SDS, 150 mM NaCl, 2 mM EDTA, 20 mM Tris-Cl) and three times with high salt wash buffer (1% (v/v) triton X-100, 0.1% (w/v) SDS, 500 mM NaCl, 2 mM EDTA, 20 mM Tris-Cl). Captured FH was eluted from the beads by incubation with elution buffer (0.1 M glycine pH 2-3) for 10 minutes on a rocking platform. The reaction was neutralized with neutralization buffer (1 M Tris-HCl, pH 8.0) which was added 1:1 to the eluate. To increase the yield of the precipitation, the unbound fraction was subjected to two more immunoprecipitation rounds following the same procedure. Purified FH was pooled and stored at -80°C until further use. Presence and concentration of FH in the eluate was determined using ELISA as described previously (21).

To confirm specificity of the FH immunoprecipitation, a precipitation was performed using 12.5% (v/v) FH-depleted serum (CompTech) with OX-24-coupled beads and 12.5% (v/v) NHS with beads coupled to human immunoglobulins (30 µg Human IgG, Sigma-Aldrich).

### 2.6 Functional factor H assays

We deployed two functional assays to assess the functionality of our FH samples of interest, i.e. FH immunoprecipitated from SP-HUS patient sera, NHS, and Neu-NHS, and of the *in vitro* dFH generated following the protocol of Gómez Delgado et al. (16).

#### 2.6.1 C3b degradation assay

To evaluate FH’s cofactor activity, we conducted a C3b degradation assay. Immunoprecipitated FH proteins were diluted to a final concentration of 1.6 µg/mL in the reaction in ice-cold Tris-buffered saline (TBS, 50 mM Tris-HCl, 150 mM NaCl, pH 7.4). C3b (7.5 µg/mL in the reaction, CompTech) and FI (1 µg/mL in the reaction, CompTech) were added to the diluted FH proteins in a final volume of 25 µL and incubated for 2 hours at 37°C. As controls, immunoprecipitated FH from NHS (wild type FH) and a condition without the addition of FH (negative control) were included. C3b degradation was analyzed by detecting formed iC3b with ELISA as described previously (29).

#### 2.6.2 Hemolytic assay

To assess FH’s ability to regulate the AP on sheep erythrocytes, we performed a hemolytic assay that was described previously (30). Sheep erythrocytes (Hatunalab) were washed two times with AP-CFTD (2.5 mM barbital, 1.5 mM sodium barbital, 144 mM NaCl, 7 mM MgCl_2_, and 10 mM EGTA, pH 7.2–7.4) by centrifugating the erythrocytes at 800*xg* for 2 minutes at 4°C. The erythrocytes were resuspended in AP-CFTD and 2.7 µL Amboceptor (Siemens) was added to the suspension after which the erythrocytes were incubated for 30 minutes at 37°C and 600 rpm. Following incubation, the sensitized erythrocytes were washed three times with AP-CFTD at 800*xg* for two minutes at 4°C, and subsequently resuspended in AP-CFTD. The sensitized erythrocyte suspension (containing 1.0-1.5 x10^8^/mL sheep erythrocytes) was put on ice until further use. For the reaction, NHS was diluted to a concentration of 12.5% serum in AP-CFTD In a V-shaped 96-wells plate (Greiner), 20 µL diluted serum was supplemented with 1 µL of 0.5 µg/µL anti-FH antibody (OX-24, Bio-Rad) per well. The plate was incubated for 15 minutes on ice to block endogenous FH. Subsequently, 5 µL of either immunoprecipitated or commercially available FH (CompTech) were added in concentrations as indicated in the figure legends. For the evaluation of immunoprecipitated samples, a starting dilution of 4 µg/mL was used. For the evaluation of *in vitro* desialylated FH prepared using the previously described protocol of Gómez Delgado et al. (16), FH was diluted to a starting dilution of 52 µg/mL. Lastly, 20 µL of the sensitized sheep erythrocytes were added to the well and the mixture was incubated for 1 hour at 600 rpm, 37°C. The reaction was halted by addition of 156 µL VBS-EDTA (2 mM barbital, 1.5 mM sodium barbital, 144 mM NaCl, and 2 mM EDTA, pH 7.4) to each well, and the plate was spun down at 1000*xg* for 2 minutes at 4°C. 180 µL of the supernatant was transferred into a new 96-wells plate and the hemolysis was assessed by measuring the absorbance at 414 nm using the Tecan Spark Spectrophotometer. As a negative control, a condition with the addition of 5 µL AP-CFTD containing no FH to the hemolytic assay reaction was included.

To evaluate the effect of neuraminidase activity in the hemolytic assay, 10 µL *C. welchii* neuraminidase (1U/mL, Sigma) was added to 10 µL of the starting dilution of the immunoprecipitated dFH condition. Subsequently, the procedure for the hemolytic assay was followed.

### 2.7 Neuraminidase activity assay

Neuraminidase activity in immunoprecipitated FH, corresponding unbound fractions, and the supernatant of the hemolytic assay was assessed using fluorometry. To the samples, 15 µL substrate (0,45 mM 2’-(4-methylumbelliferyl)-α-D-N-acetylneuraminic acid, Sigma) was added 1:1 and incubated for 2 hours at 37°C. After incubation, the reaction was stopped by addition of 200 µL stop buffer (0,8 M glycine, 0,8 mM NaCl, 0,025% Triton X-100 (w/v), pH 10,6). As a blank measurement, the substrate was incubated without sample, and 15 µL sample was added once the incubation was finished. We then prepared the standard and standard blank. For this, 15 µL of the standard (18 µM 4-methylumbelliferone (Sigma) in 0,1 M HCl) was mixed with 15 µL milli-Q water and 200 µL stop buffer. For the standard blank measurement, 30 µL milli-Q water was mixed with 200 µL stop buffer. All samples were measured in duplicate and the standard and standard blank were measured in triplicate. Fluorescence was measured with the Tecan Spark spectrophotometer, at 360 nm excitation and 446 nm emission.

### 2.8 Data analysis and statistics

Data are presented as mean ± standard deviation (SD). Data visualization and statistical calculations were performed using GraphPad Prism version 10 for Windows. Comparisons between multiple groups were analyzed with the one-way ANOVA with Dunnett’s multiple comparison test. Comparisons between columns and rows were analyzed with a multiple unpaired t-test with Holm-Šídák’s multiple comparison test. Principal component analysis (PCA) was performed using MetaboAnalyst 6.0 (31) with mean centered data.

## 3 Results

### 3.1 Glycopeptide profile of factor H in healthy controls

**Figure 1:**
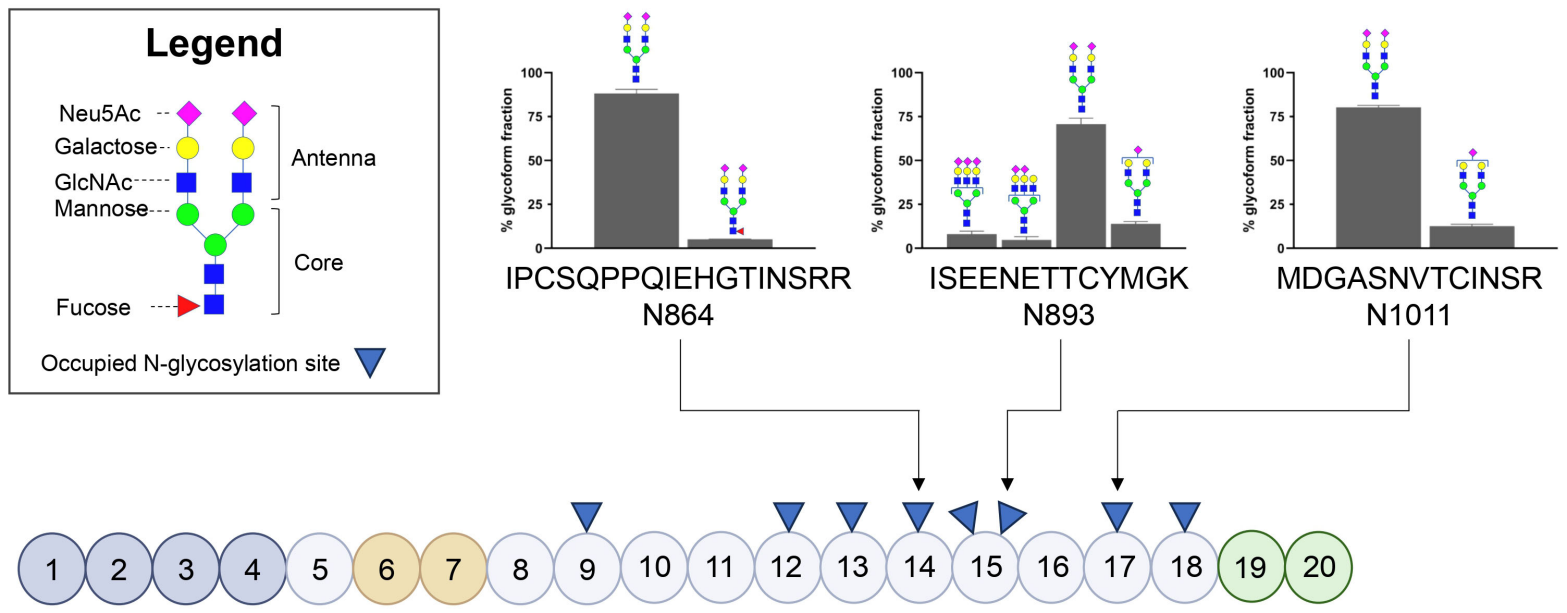
Glycosylation status of Factor H in healthy controls based on three glycopeptides. Schematic representation of Factor H (FH) illustrating its Short Consensus Repeats (SCR) domains and the locations of occupied N-linked glycan sites (indicated with blue triangles). Domains involved in C3b binding (SCR1-4) are highlighted in dark grey, heparin-binding domains (SCR6-7) are highlighted in orange, and domains involved in sialic acid, heparin and C3b binding (SCR19-20) are highlighted in green. The figure was adapted from Schmidt et al. (32). N-glycosylated asparagine (N) residues analyzed during this study are annotated using arrows. The bar graphs display the distribution of the N-glycans present on the three glycopeptides in three healthy controls, with values representing the mean presence of the glycoform over three healthy controls ± SD as a percentage of the total glycoform fraction. Glycoforms present ≤4% are not shown.

FH contains eight occupied N-glycosylation sites, divided over short consensus repeat (SCR) domains SCR9, SCR12, SCR13, SCR14, SCR15 (two N-glycosylation sites), SCR17 and SCR18; notably, these sites cluster outside of the ligand-binding domains (**Figure 1**). Using an established LC-MS/MS-based N-glycoproteomics approach, we reliably detected three glycopeptides of FH located in SCR14 (IPCQPPQIEHGTINSRR, further referred to as N864), SCR15 (ISEENETTCYMGK, further referred to as N893) and SCR17 (MDGASNVTCINSR, further referred to as N1011). Across these glycosylation sites, we confirmed the presence of bi-antennary mature N-glycans (Hex5HexNAc4NeuAc2, abbreviated as H5N4S2) at all three sites, with a minor presence of tri-antennary glycans (H6N5S3 and H6N5S2) located at N893. Additionally, we observed low levels of fucosylation (H5N4F1S2) of the bi-antennary glycans located at N864 and a minor presence of bi-antennary glycans lacking one sialic acid (H5N4S1) at N893 and N1011 (**Figure 1**). Our findings are consistent with reference data, confirming that these sites are occupied by N-linked glycans (27), and are further supported by previous reports describing similar glycan moieties on FH (17, 19).

### 3.2 Glycopeptide profile of factor H in SP-HUS patients during the acute phase and remission

**Figure 2:**
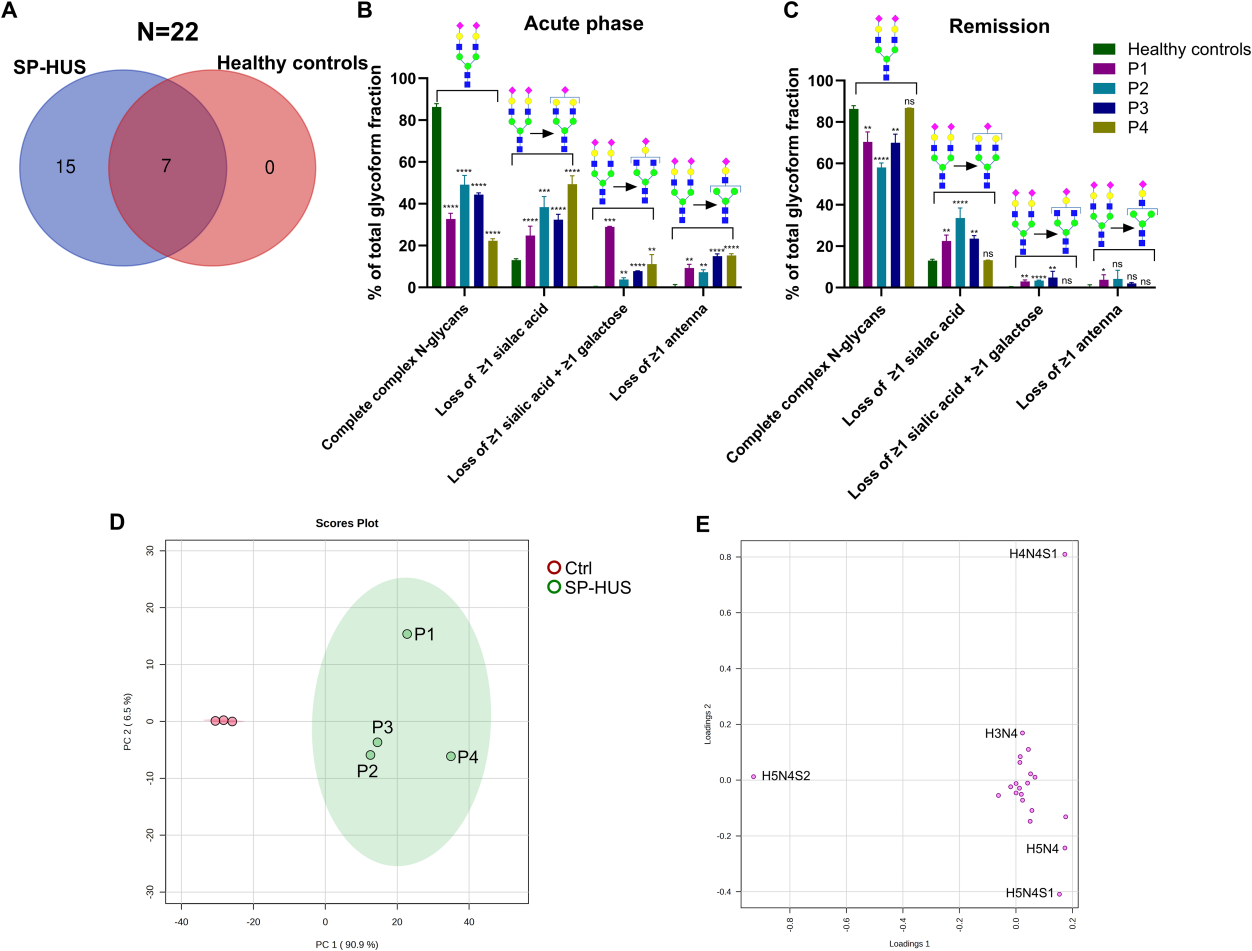
Glycosylation status of Factor H in SP-HUS patients. **(A)** Venn diagram summarizing the identified N-glycoforms on three Factor H (FH) glycopeptides (N864, N893 and N1011) using LC-MS/MS glycopeptide profiling. In total, 22 distinct N-glycan structures were identified: 15 unique to SP-HUS patients during the acute phase, 7 shared between SP-HUS patients and healthy controls, and none unique to healthy controls. **(B, C)** Comparative analysis of glycosylation changes during the acute phase **(B)** and remission **(C)** in SP-HUS patients versus healthy controls. LC-MS/MS data were interpreted using predefined glycan groups: complete complex N-glycans, loss of ≥1 sialic acid, loss of ≥1 galactose and loss of ≥1 antenna (consisting of at least 1 sialic acid, 1 galactose and 1 GlcNAc). Analysis includes N-glycans detected at glycopeptide sites N864, N893 and N1011. Schematic representations of a representative glycoform for each category is shown. Full details of glycan subgroups are provided in the **Supplementary Figure 2**. Data is shown as mean ± SD of two measurements. Statistical significance was determined using a multiple unpaired T test followed by Holm-Šídák’s multiple comparison test comparing the patient values to the control values: ns, not significant; * p ≤ 0.5; ** p ≤ 0.01; *** p ≤ 0.001 and **** p ≤ 0.0001. **(D, E)** Principal component analysis (PCA) of FH glycopeptide profiling data during the acute phase of SP-HUS patients (SP-HUS) and in healthy controls (Ctrl). The score plot **(D)** demonstrates clear separation in both the first and second principal component and no overlap of the 95% confidence intervals. The loading plot **(E)** indicates that separation of the SP-HUS group is primarily driven by glycans lacking sialic acid (e.g., H5N4 and H5N4S1), galactose (H4N4S1) and a complete antenna (H3N4). Corresponding Scree plot of PCA analysis can be found in the **Supplementary Material, Figure 4**.

Next, we investigated the glycosylation status of FH in patients with SP-HUS. We evaluated the samples of four SP-HUS patients during the acute phase and remission, and compared the measurements to three healthy controls. We identified 22 distinct N-glycans on the three FH glycopeptides in SP-HUS patients. Fifteen of these N-glycans were only seen in the evaluated SP-HUS patients during the acute phase, the other seven N-glycans were observed in healthy controls as well (**Figure 2A**).

The changes in glycans that we observed in SP-HUS patients were grouped based on glycan traits (see **Supplementary Figure 2** for schematic representations of the glycans included in each subgroup). In all four SP-HUS patients, we observed a significant decrease in the mature complete N-glycans with two or more antenna during the acute phase, resulting in a higher presence of N-glycans lacking one or more sialic acids compared to the healthy controls. Surprisingly, we also observed a significant appearance of glycoforms with ≥1 galactose loss in addition to the loss of ≥1 sialic acid, and appearance of even further truncated N-glycans with a complete loss of ≥1 antenna in the acute phase for all SP-HUS patients (**Figure 2B**). These data therefore show that there is not only a loss of sialic acids on FH during the acute phase of SP-HUS, but also of other sugar moieties and even complete antennae on FH.

In the remission of SP-HUS, we observed complete normalization for P4, and an improvement, but not a complete normalization, of the N-glycosylation profile on FH for P1, P2 and P3. In these four patients, the presence of the mature complete N-glycans increased to a mean of 71% of the total glycoform fractions (*vs* 37% in the acute phase and 86% in healthy controls) and we observed a decrease of the truncated glycans associated with loss of sialic acids (6% in remission *vs* 36% in the acute phase), galactose (3% in remission *vs* 11% in the acute phase) and complete antennae (2% in remission *vs* 12% in the acute phase) (**Figure 2C**, **Supplementary Table 3**).

PCA was employed to explore the variance between the healthy controls and SP-HUS patients during the acute phase. We observed a clear separation of controls and SP-HUS patients in both the first and second principal component with no overlap between the 95% confidence intervals (**Figure 2D**). The loading plot from the PCA analysis confirms that the separation of the SP-HUS group *vs* control group is based on truncated glycans lacking e.g. sialic acid (H5N4 and H5N4S1), galactose (H4N4S1) and a complete antenna (H3N4) in the SP-HUS group. In the controls, the presence of the di-antennary glycoform (H5N4S2) is separating the control group from the SP-HUS patient group (**Figure 2E**, see **Supplementary Figure 5** for corresponding scree plot).

**Figure 3:**
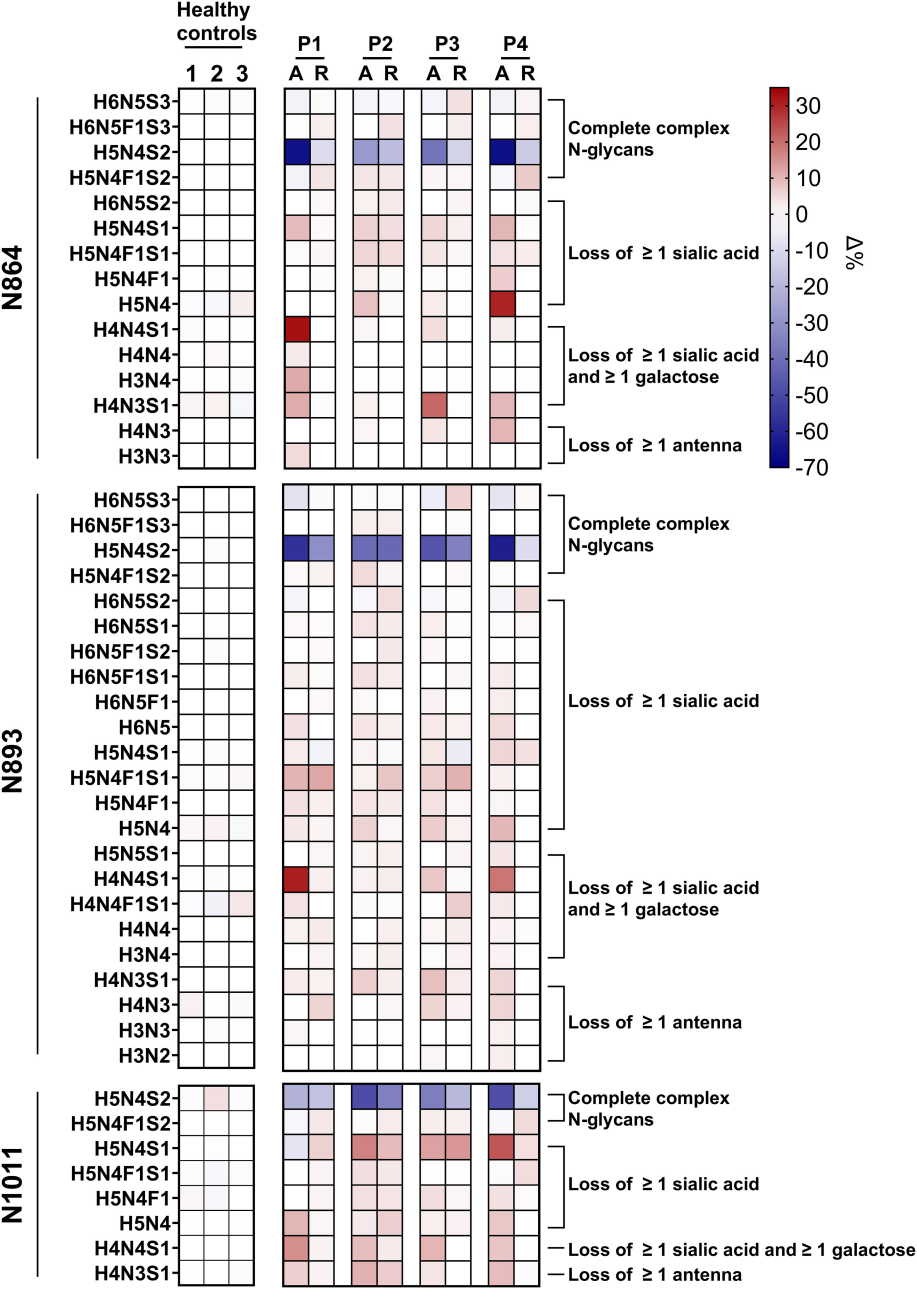
Heatmap of Factor H glycopeptide profiling in SP-HUS patients and healthy controls. The glycosylation profiles of three Factor H (FH) glycopeptides (IPCSQPPQIEHGTINSSR, N864; ISEENETTCYMGK, N893; and MDGASNVTCINSR, N1011) are shown for four SP-HUS patients and healthy controls. Data from three healthy controls were averaged and used as a reference to determine the Δ% of the glycoform fraction in SP-HUS patients compared to the controls. For each SP-HUS patient (P1-P4), samples from both the acute phase (A) and remission (R) are represented. Glycosylation changes are expressed as Δ%, where red cells depict an increase, and blue cells depict a decrease in the abundance of the respective glycoform relative to the controls. Values represent the mean of two measurements.

When looking in more detail at the glycosylation changes at the level of each glycan on each glycopeptide individually, it is apparent that all SP-HUS patients show significant N-glycosylation changes across all three sites (N864, N893 and N1011), with a particular loss of the mature di-sialo N-glycans (H5N4S2, **Figure 3**, **Supplementary Figure 3**). Next to a reduction of the mature complex N-glycans, we observe an increase of glycoforms lacking ≥1 sialic acid, ≥1 galactose, and complete antennae. While we detect severe changes in N-glycosylation on FH in all patients, we also observed differences between patients and N-glycosylation sites. For example, P1 and P4 seem to be affected more severely with regards to N-glycosylation loss, showing a severe decrease of bi-antennary N-glycans capped with two sialic acids (H5N4S2) at sites N864 and N893, where P2 and P3 appear less severely afflicted. Furthermore, in P1 and P4, we exclusively detected the presence of the (partial) N-glycan core structure (H3N3 and H3N2) at glycosylation sites N864 and N893. In P1 we detected an increase of 30% of glycoform H4N4S1 in the acute phase at sites N864 and N893, while this glycoform was not detected to this extent in the other patients and healthy controls. Similarly, P4 shows a strong increase of the glycoform H5N4 at site N864 while this glycoform was detected to a lesser extent in P1-P3. Interestingly, we observed loss of sialic acids to a lesser extent on site N1011 when compared to N864 and N893 for P1 and P4. While P2 and P3 appear less afflicted with regards to the sialic acid loss, site N864 and N1011 appear to have retained more of the sialic acids when compared to site N893.

### 3.3 Functional evaluation of desialylated factor H obtained from neuraminidase treated serum

To evaluate the functional consequence of desialylation of FH, we treated NHS with *C. welchii* neuraminidase (Neu-NHS) *in vitro*. Using LC-MS/MS glycopeptide profiling, we showed a complete loss of sialic acids on three glycopeptides (N864, N893 and N1011) of FH after neuraminidase treatment compared to the control sample that had not been subjected to neuraminidases (**Supplementary Figure 4**). Other sugar moieties such as galactose and N-acetylglucosamine (GlcNAc) were not affected by this treatment. Therefore, we concluded that the creation of desialylated FH (dFH) with neuraminidase was successful.

To eliminate the neuraminidases from Neu-NHS for functional analysis, we subsequently isolated dFH with immunoprecipitation using OX-24 coupled beads. We first validated our immunoprecipitation protocol to show that this method was effective for FH purification (**Supplementary Table 4**). Following immunoprecipitation of the Neu-NHS sample, FH levels were determined in the sample eluates using ELISA and absence of neuraminidase activity in the eluate sample was confirmed using a fluorometry-based neuraminidase activity assay (**Supplementary Table 5**).

**Figure 4:**
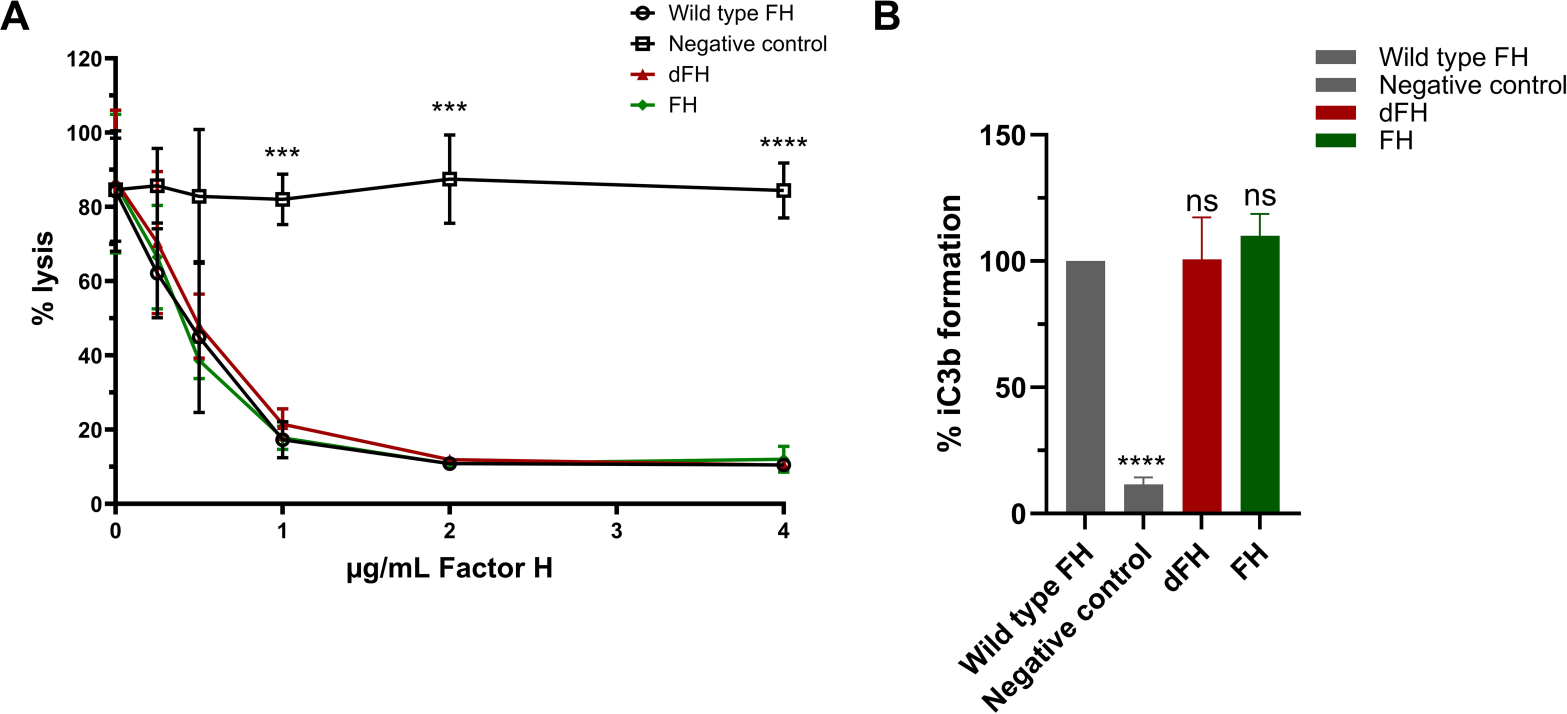
Functional analysis of *in vitro* desialylated Factor H obtained from neuraminidase-treated serum. **(A)** Sheep erythrocyte hemolytic assay evaluating the complement regulatory activity of Factor H (FH) to bind and protect the erythrocytes from complement-mediated lysis. FH was isolated by immunoprecipitation from Normal Human Serum (NHS; wild type FH), NHS treated with neuraminidase (desialylated FH; dFH), and NHS treated in parallel without neuraminidase (control FH; FH) was added to NHS treated with OX-24 in the concentrations indicated on the x-axis. A negative control without the addition of FH was included. Hemolysis is depicted as % of full lysis of erythrocytes in water. The values are shown as mean ± SD of three experiments. Statistical comparisons to wild type FH were performed using multiple unpaired T tests followed by Holm-Šídák’s multiple comparison test; *** p ≤ 0.001 and **** p ≤ 0.0001. **(B)** FH cofactor activity assessing the generation of iC3b from C3b by Factor I (FI) in presence of FH using ELISA. The data are presented as % iC3b formation normalized to wild type FH. As controls, FH isolated from NHS (wild type FH) and a C3b degradation without the addition of FH (negative control) were included. The values are shown as mean ± standard deviation of three experiments. Samples were compared to wild type FH using one-way ANOVA followed by Dunnett’s multiple comparison test. ns, not significant; ****p ≤0.0001.

Next, we tested the *in vitro* dFH in two functional assays. The hemolytic assay using sheep erythrocytes evaluated the regulatory ability of FH to bind and protect sheep erythrocytes from complement-mediated hemolysis. The FH samples were added in increasing conditions to NHS in which the endogenous FH was blocked by addition of a high-affinity monoclonal anti-FH antibody prior to starting the assay. The C3b degradation assay assessed the cofactor activity of FH to aid in the cleavage of C3b to iC3b by FI. Interestingly, we found no functional difference between *in vitro* dFH obtained from Neu-NHS and wild type FH which was isolated from NHS not treated with neuraminidase in both the hemolytic assay and C3b degradation assay (**Figure 4**). This suggests that while the sialic acids from FH’s N-glycans were removed, FH retained its complement-regulatory functions.

### 3.4 Functional evaluation of SP-HUS patient factor H

**Figure 5:**
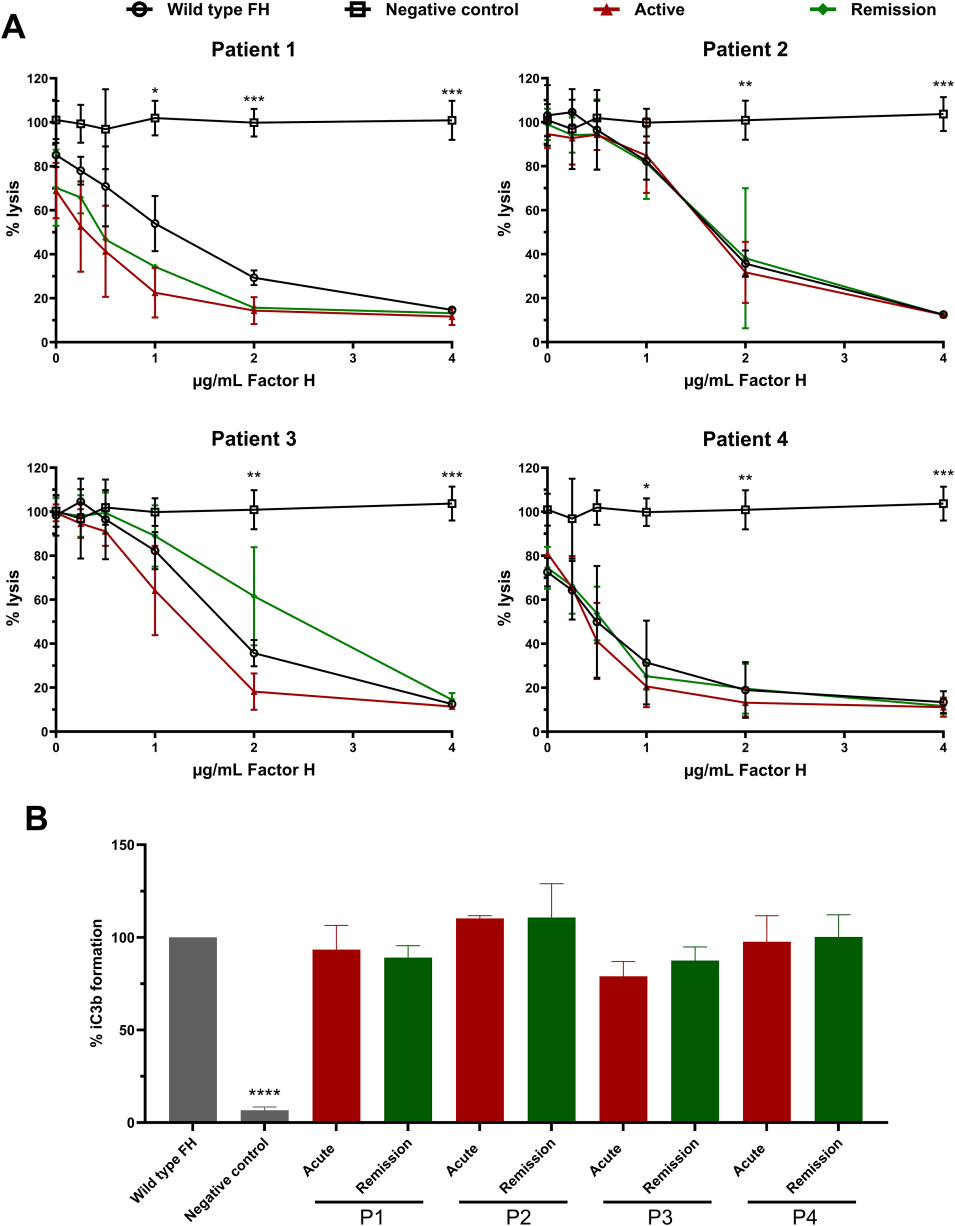
Functional analysis of Factor H obtained from SP-HUS patient material. **(A)** Sheep erythrocyte hemolytic assay evaluating the complement regulatory activity of Factor H (FH) to bind and protect the erythrocytes from complement-mediated lysis. FH was isolated by immunoprecipitation from Normal Human Serum (NHS; wild type FH), and SP-HUS patient sera (P1-P4) during the acute phase and remission. FH was added to NHS treated with OX-24 in the concentrations indicated on the x-axis. A negative control without the addition of FH was included. Hemolysis is depicted as % of full lysis of erythrocytes in water. The values are shown as mean ± SD of three experiments. Statistical comparisons to wild type FH were performed using multiple unpaired T tests followed by Holm-Šídák’s multiple comparison test; * p ≤ 0.05; ** p ≤ 0.01; and *** p ≤ 0.001. **(B)** FH cofactor activity assessing the generation of iC3b from C3b by Factor I (FI) in presence of FH using ELISA. The data are presented as % iC3b formation normalized to wild type FH. As controls, FH obtained from NHS (wild type FH) and a C3b degradation without the addition of FH (negative control) were included. The values are shown as mean ± SD of three experiments. Samples were compared to wild type FH using one-way ANOVA followed by Dunnett’s multiple comparison test; ****p ≤0.0001.

Since we observed that the N-glycans of FH in SP-HUS patients show glycosylation alterations beyond desialylation (**Figures 2**, **3**, **Supplementary Figure 3**), we evaluated the functionality of FH immunoprecipitated from SP-HUS patient samples and compared it to the wild type. FH isolated from SP-HUS patients and wild type FH both were able to protect the sheep erythrocytes from hemolysis in a dose-dependent manner (**Figure 5A**). No significant differences were detected between FH isolated from SP-HUS patients (both the acute phase and remission) and wild type FH (**Figure 5A**). When we assessed the cofactor activity of SP-HUS patient FH, we observed similar results. FH from all four SP-HUS patients obtained during the acute phase and remission was able to aid in C3b degradation, with no statistical difference with wild type FH (**Figure 5B**). These results imply that the altered glycosylation status of FH during SP-HUS does not affect the complement-regulatory function of FH itself.

### 3.5 Further evaluation of *in vitro* desialylated factor H

**Figure 6:**
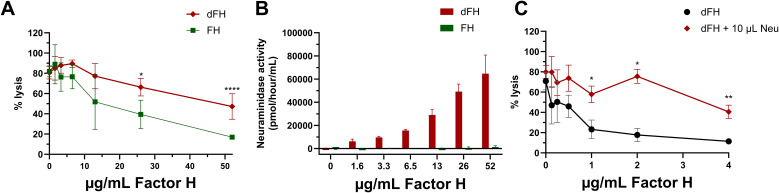
Evaluation of *in vitro* desialylated Factor H and neuraminidase activity. **(A)** To evaluate the *in vitro* desialylation of commercially available Factor H (FH) we prepared *in vitro* desialylated FH (dFH) using the Gómez Delgado protocol (16), with the inclusion of a control sample without neuraminidase (FH). FH functionality was evaluated using a sheep erythrocyte hemolytic assay evaluating the ability of FH to bind and protect the erythrocytes from complement-mediated lysis. FH and dFH were added to NHS treated with OX-24 in the concentrations indicated on the x-axis. Hemolysis is depicted as % of full lysis of erythrocytes in water. The values shown are presented as mean ± SD of three preparations of dFH and FH, each measured in two separate experiments. Statistical comparisons between dFH and FH were performed using multiple unpaired T tests followed by Holm-Šídák’s multiple comparison test; * p ≤ 0.05; and **** p ≤ 0.0001. **(B)** Fluorometry-based neuraminidase activity assay performed the supernatant of the hemolytic assay of FH and dFH samples prepared with the Gómez Delgado protocol. The x axis depicts the concentration of dFH/FH which was added into each well. The values are shown as mean ± SD of three separate experiments. **(C)** Effect of the presence of neuraminidase in the hemolytic assay using immunoprecipitated desialylated FH from NHS treated with neuraminidase (dFH). An additional amount of neuraminidase (Neu; 10 µL) was added into the start dilution of the dFH sample. Hemolysis is depicted as % of full lysis of erythrocytes in water. Data is shown as mean ± SD of three separate experiments. The sample containing additional neuraminidase (dFH + 10 µL Neu) was compared to the condition without neuraminidase (dFH) using a multiple unpaired T test followed by Holm-Šídák’s multiple comparison test; * P ≤ 0.5 and ** p ≤ 0.01.

Our findings on the regulatory capacity of dFH on erythrocytes were in contrast to a previously published report (16), and this prompted us to further investigate potential differences in the experimental procedures that were used. To explore this, we desialylated FH *in vitro* following the same protocol reported by Gómez Delgado et al. (16), by treating commercially obtained serum-purified FH with neuraminidase instead of immunoprecipitating FH from Neu-NHS. After the *in vitro* desialylation of FH, the neuraminidase activity was stopped by addition of a stop buffer (pH 10) with no additional steps to remove neuraminidases from the sample. When testing these dFH samples in the hemolytic assay, we found increased hemolysis when compared to FH not treated with neuraminidase (**Figure 6A**). This is in line with the findings of Gómez Delgado et al., but in contrast with our own findings of dFH (**Figure 4A**). However, we suspected that the increased lysis was not due to loss of FH functionality but due to residual neuraminidase activity in the dFH. Indeed, when collecting supernatant samples of the hemolytic assay, we detected a pH of 7.0 (data not shown) and subsequent fluorometry analysis revealed neuraminidase activity in the supernatant samples of the hemolytic assay in a dose-dependent manner (**Figure 6B**). To further confirm that neuraminidase activity affects lysis of the erythrocytes in the hemolytic assay, we added 10 µL 1 U/mL *C. welchii* neuraminidase to our own immunoprecipitated dFH sample, and showed increased hemolysis of the erythrocytes (**Figure 6C**). This effect is likely due to the cleavage of sialic acids of the erythrocyte surface, which results in sensitization for complement-mediated hemolysis (33).

Of note, during these experiments it was observed that serum-purified FH from CompTech is less biologically reactive than immunoprecipitated FH. Specifically, FH from CompTech required a starting dilution up to 52 µg/mL FH to inhibit lysis of the erythrocytes, while immunoprecipitated FH only requires up to 4 µg/mL (**Figures 6A, C**).

## 4 Discussion

SP-HUS is a rare and severe complication arising from an invasive *S. pneumoniae* infection. Compared to other forms of HUS, SP-HUS has a more severe disease course with a mortality rate reported of 12%. Notably, while only a minority of patients presents with meningitis, this group accounts for 88% of reported deaths (6, 34, 35). Despite the severity of the disease, the pathophysiology of SP-HUS remains poorly understood, and research on the disease is limited. Furthermore, currently there are no targeted therapies available, and management relies on supportive treatments such as antibiotics and dialysis. Although many aspects of the involvement of the complement system in the pathophysiology of SP-HUS remain unclear, it is hypothesized that desialylation of FH by neuraminidases during SP-HUS may contribute to disease pathogenesis. Recent work has shown desialylation of FH in SP-HUS patients during the acute phase, and *in vitro* desialylated FH resulted in decreased regulatory activity of FH on sheep erythrocytes (16). Therefore, we aimed to investigate the glycosylation status of FH during the acute phase and remission in four SP-HUS patients and evaluated the complement regulatory function of FH purified from SP-HUS patient serum during the acute phase and remission.

Our findings using high-resolution LC-MS/MS-based N-glycoproteomics reveal significant alterations in FH glycosylation during the acute phase of all four SP-HUS patients compared to healthy controls. Interestingly, N-glycosylation changes on FH were not limited to desialylation. We are the first to describe that the glycosylation changes also include loss of galactose and N-acetylglucosamine (GlcNAc) residues (**Figure 2**). When we examined the individual patients and glycosylation sites, we observed that all three examined N-glycosylation sites are affected. Furthermore, P1 and P4 were more severely affected with regards to N-glycosylation changes on these three glycopeptides, while P2 and P3 exhibited milder alterations (**Figure 3**). The observed differences in glycosylation between individual patients could be due to factors such as disease severity, variation in timing of the blood sampling, disease progress, or variability in the bacterial strain involved in infection. Of note, while in P3 SP-HUS infection could not be proven, and genetic screening is lacking, the clinical manifestation strongly supports the SP-HUS diagnosis and not CaHUS. Furthermore, the glycosylation changes of FH in P3 strongly resembled those seen in P2, in which SP-HUS was confirmed. While no differences in long-term renal outcomes were observed for these patients, P1 and P4 did exhibit differences in disease manifestations when compared to P2 and P3 (**Supplementary Table 1**). Specifically, P1 and P4 required longer hospitalizations (31 and 47 days, respectively) compared to P2 and P3 (23 and 13 days). P4 had a longer need for dialysis (29 days), followed by P1 (8 days), P3 (4 days), and P2 (no need for dialysis). Additionally, P1 and P4 had a greater need for erythrocyte transfusions (7 and 6 units, respectively) compared to P2 and P3 (both 3 units). These findings may suggest that the more pronounced N-glycosylation changes detected in P1 and P4 in the acute phase may be associated with a more severe acute disease course, despite similar long-term outcomes among all patients. Given the limited sample size of four SP-HUS patients and the focus on the N-glycan profile of FH in our study, additional studies involving larger cohorts and a wider N-glycosylation profile comprising more proteins are necessary to substantiate these findings and clarify the relationship between N-glycosylation patterns and disease severity in SP-HUS.

In remission, the glycosylation patterns of the SP-HUS patients improved, but they did not fully normalize in P1, P2, and P3. We observed an increase of the complete complex N-glycans, and a decrease of the glycans associated with loss of sialic acid, galactose, and complete antennae when compared to the acute phase. In the remission sample of P4, the N-glycosylation pattern on FH was completely normalized and comparable to the healthy controls (**Figures 2**, **3**). Remission samples were collected when the patients reached hematological remission, with a short interval between samples for P2 (11 days after acute phase) and P3 (15 days after acute phase) (**Supplementary Table 2**). P1 and P4 had a longer interval between the acute phase and remission samples (118 and 135 days after acute phase, respectively). We hypothesize that the glycopeptide profile of FH reflects lingering effects of the SP-HUS episode at the protein level, even after hematological remission has been achieved. FH has a half-life of approximately 6 days (36) and the abnormally glycosylated forms of FH could still be present in the circulation several weeks after disease onset. Currently, there is limited data on the stability of bacterial neuraminidases *in vivo*, particularly during the course of treatment. One study reported the absence of detectable neuraminidase activity in an SP-HUS patient by day 3 of treatment (37). However, it is possible that in other patients neuraminidases are still present in circulation during treatment. Further studies should assess neuraminidase activity in SP-HUS patient sera at various disease stages to better understand its persistence and role in disease progression.

FH levels measured during the acute phase indicated that P1 had moderately decreased FH levels just below the control range. In remission, FH levels for all four patients increased when compared to the corresponding acute phase, and were within the normal range for all patients (**Supplementary Figure 1**). This is consistent with an earlier report, where SP-HUS patients showed decreased levels of complement proteins such as C3, C4, FI and FH (38). This decrease in complement proteins might be due to complement consumption, as a result of excessive complement activation induced by the complement-evading strategy of *S. pneumoniae.* The bacterium expresses several surface proteins, including PspA and PspC, which promote FH binding and inhibit complement deposition (39, 40). In addition, the bacterial toxin pneumolysin acts as a decoy by activating the classical pathway of the complement system away from the bacterial surface, diverting the complement attack. Pneumolysin thereby also contributes to complement consumption, further reducing the effective complement components available to target the bacteria (39, 41).

While desialylation of the plasma proteins transferrin and proteins in the FH family, including FH, Factor H-like 1 (FHL-1) and Factor H Related (FHR) 1-5, have been previously described in SP-HUS patients, further processing of N-glycans, including loss of galactose and GlcNAc as we have seen in our patients, has not been reported (16, 37). Many microbes, including *S. pneumoniae*, express proteins that are involved in the metabolism of glycans, as these are an important source of nutrients during growth *in vivo* (42, 43). In addition, secretion of glycosidases that modify glycans on proteins of the innate and acquired immune system, such as complement factors and immunoglobulins, might be an effective way to interfere with the host’s immune response. For example, *S. pneumoniae* has been shown to be able to process N-glycans on immunoglobulin A *in vitro* (44). We therefore think that not only the released neuraminidases were responsible for the glycosylation changes of secreted FH in the blood stream during the invasive *S. pneumoniae* infection, but also other exoglycosidases produced by the bacterium. Of note, altered *de novo* glycan synthesis by the host during the infection cannot be ruled out. The role of posttranslational modifications of proteins, including glycosylation, during infections remains significantly understudied. A recent study using comparative glycoproteomics identified distinct glycosylation patterns in young children infected with different pathogens, highlighting how infection may specifically influence glycosylation profiles (45). Additional studies are required to further elucidate the pathways involved in glycosylation during SP-HUS. In particular, *in vitro* studies could provide valuable insights.

In this study, we also examined the functional effects of the altered glycosylation status of FH in SP-HUS on complement regulation. Interestingly, we found that FH isolated from the SP-HUS patients samples retained its complement regulatory functions, as measured by its ability to act as a cofactor for FI in C3b degradation and by its ability to protect sheep erythrocytes from complement-mediated hemolysis (**Figure 5**). This was also the case for dFH purified from serum treated with neuraminidase (**Figure 4**). While FH is heavily glycosylated, its N-glycosylation sites are not subject to strict evolutionary genetic conservation and are mainly clustered in regions that are not directly involved in ligand binding (**Figure 1**) (14). It has been suggested that the glycans on FH serve predominantly indirect purposes, such as maintaining solubility or providing resistance to proteolytic degradation rather than directly affecting its complement regulatory function (14, 17). In support of this, recombinant FH produced in the moss *Physcomitrium patens* carrying either truncated N-glycans or only the core GlcNAc residue retains normal complement regulatory activity (19, 32, 46). While the hemolytic assay we employed provides an indirect readout of total FH regulatory activity, it does not assess all functionalities of FH separately. For example, we were unable to assess the ability of FH to specifically decay C3 convertases. However, previous studies have demonstrated that the removal of FH’s N-glycans is unlikely to significantly impair this specific function. FH carrying desialylated and truncated N-glycans, and FH containing only the core GlcNAc residue had a normal ability to decay cell-bound C3-convertases *in vitro* (18, 19, 32). Collectively, our findings suggest that extensive loss of N-glycan structures on FH during SP-HUS episodes does not affect its canonical complement-regulatory function and that specific FH deglycosylation on itself is unlikely to contribute to disease pathogenesis through dysregulation of the AP.

Nonetheless, our findings and previously reported results (18, 19, 32) were contradictory with those reported by Gómez Delgado et al., who showed decreased AP control by *in vitro* desialylated FH in a similar sheep erythrocyte hemolytic assay as we used (16). When we repeated their reported protocol to obtain dFH, i.e. by treating FH with neuraminidase and stopping the reaction with a high pH, and tested it in our assay, we also detected increased hemolysis of the sheep erythrocytes. However, we also found neuraminidase activity in the supernatants of the hemolytic assay (**Figure 6**). Given that *C. welchii* neuraminidases have an optimal enzyme activity at a pH of 5.5 and are inhibited at pH levels above 9 (47, 48), we hypothesize that inhibition by addition of a high pH stop buffer to the neuraminidase-treated sample may not be permanent and can thus be reversed when the pH of the sample is changed. Importantly, desialylation of sheep erythrocytes is known to enhance their susceptibility to complement-mediated lysis, since they lose their ability to recruit FH to protect them against complement attack (33). We therefore speculate that the results reported by Gómez Delgado et al. may reflect enhanced erythrocyte vulnerability for complement attack due to neuraminidase activity, rather than impaired regulatory function of FH. However, we cannot rule out the possibility that desialylation of other complement factors or plasma proteins may have contributed to the observed hemolysis. We would like to highlight that our immunoprecipitation protocol to purify dFH from neuraminidase-treated serum circumvents possible issues with residual neuraminidase activity.

In this study, we focused on the well-characterized functions of FH in regulating the AP. However, in recent years additional and less well-studied functions of FH have been elucidated, including its interaction and competition with different members of the FH protein family (FHL-1 and CFHR1-5) in fine-tuning AP activation and regulation (49, 50). We have not specifically studied how FH glycosylation changes might influence these competitive dynamics, and the impact of N-glycosylation on the competition between FH and CFHR proteins for C3b binding is currently unclear. Beyond FH’s well established and well-studied role in complement regulation, FH also exhibits non-canonical functions, notably though direct binding to complement receptors CR3 and CR4 on myeloid cell types such as neutrophils, monocytes, macrophages, and epithelial cells (51). This receptor interaction modulates key cellular functions, including cell-cell contacts, proliferation, phagocytosis and transendothelial migration of immune cells (52–54). Possibly, aberrant glycosylation of FH may alter its ability to engage these receptors, potentially contributing to altered immune responses in patients with SP-HUS. Future studies should assess the specific impact of FH glycosylation on the interaction and competition with other members of the FH protein family, receptor binding, and downstream immune functions.

While we did not detect functional changes for FH, it would be of great value to evaluate the glycosylation status of other plasma proteins in relation to SP-HUS as well. Many proteins in both the innate and adaptive immunity are also highly glycosylated, and alterations of these glycans may possibly affect immune recognition, complement activation, and/or bacterial clearance (55–57). Given the observed glycosylation changes on FH, glycan-based biomarkers could be investigated as potential tools for SP-HUS diagnosis and disease monitoring in the future. Additionally, if deglycosylation-based disease drivers are recognized, targeting bacterial glycosidases or blocking glycan modification might offer novel therapeutic strategies.

In conclusion, we demonstrate extensive trimming of complex N-glycans on FH during the acute phase of SP-HUS. Notably, removal of sialic acids, galactose and N-acetylglucosamine from FH does not compromise its ability to regulate the AP, which indicates that FH deglycosylation during SP-HUS does not directly cause dysregulation of the complement system. SP-HUS remains a poorly understood disease with no targeted therapies currently available. Future research should explore the functional consequences of deglycosylation of proteins within both the innate and adaptive immunity and how these changes may relate to the development of SP-HUS to advance future treatment options.

## Data Availability

The datasets presented in this study can be found in online repositories. Mass spectrometry data and codes used in this paper are publicly available. The mass spectrometry proteomics data have been deposited to the ProteomeXchange Consortium via the PRIDE (58) partner repository with the dataset identifier PXD064837. The original codes used to process the data have been published previously (23) and have been deposited to the ProteomeXchange Consortium via the PRIDE partner repository with the dataset identifier PXD034214. Further inquiries can be directed to the corresponding author.
