# Supplementary material for: Glycoproteomics analysis of complement factor H and its complement-regulatory function during Streptococcus pneumoniae-associated hemolytic uremic syndrome
Source: Front Immunol. 2025 Aug 21;16:1645196. doi: 10.3389/fimmu.2025.1645196 (PMC12408639; doi:10.3389/fimmu.2025.1645196)
Supplement: Supplementary file 1 [file DataSheet1.docx]

Supplementary Material

Glycoproteomics analysis of Complement Factor H and its complement regulatory function during *Streptococcus pneumoniae-*associated hemolytic uremic syndrome

Laura M. Baas^1^, Kioa L. Wijnsma^1^ , Fokje Zijlstra^2^, Nicole C.A.J. van de Kar^1^, Lieke ter Steeg^1^, Antonia H.M. Bouts^3^, Marloes A.H.M. Michels^2^, Jeroen D. Langereis^4^, Dirk Lefeber^2,5^, Hans J.C.T. Wessels^2†^, Lambertus P. van den Heuvel^1,2,3,6,7†*^

*** Correspondence:**Lambertus P. van den Heuvel
bert.vandenheuvel@radboudumc.nl

# Supplementary Figures and Tables

## *
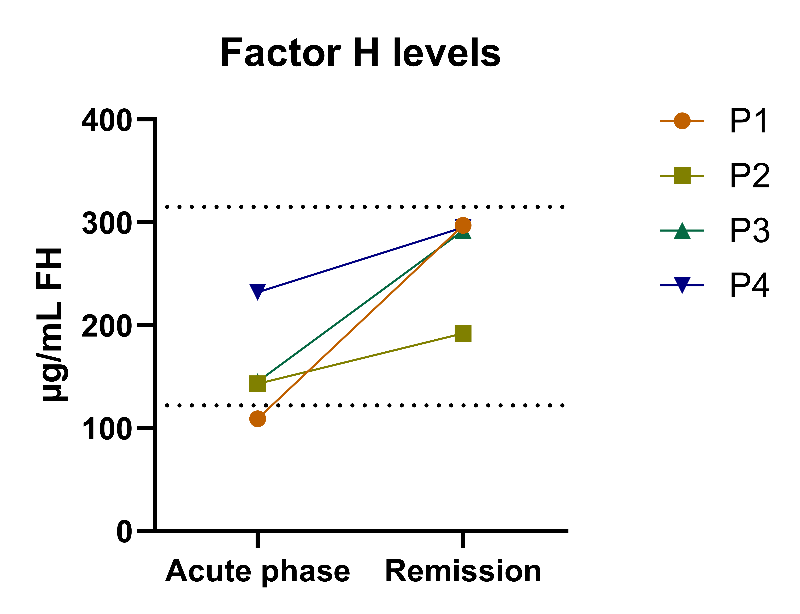
*Supplementary Figures

**Supplementary Figure 1,** **FH levels measured in SP-HUS patient samples (P1-P4) during the acute phase and remission with ELISA.** *The range of FH levels in 37 healthy controls is indicated by the dotted lines (122-315 µg/mL). Samples were measured in duplicate in two dilutions.*

**
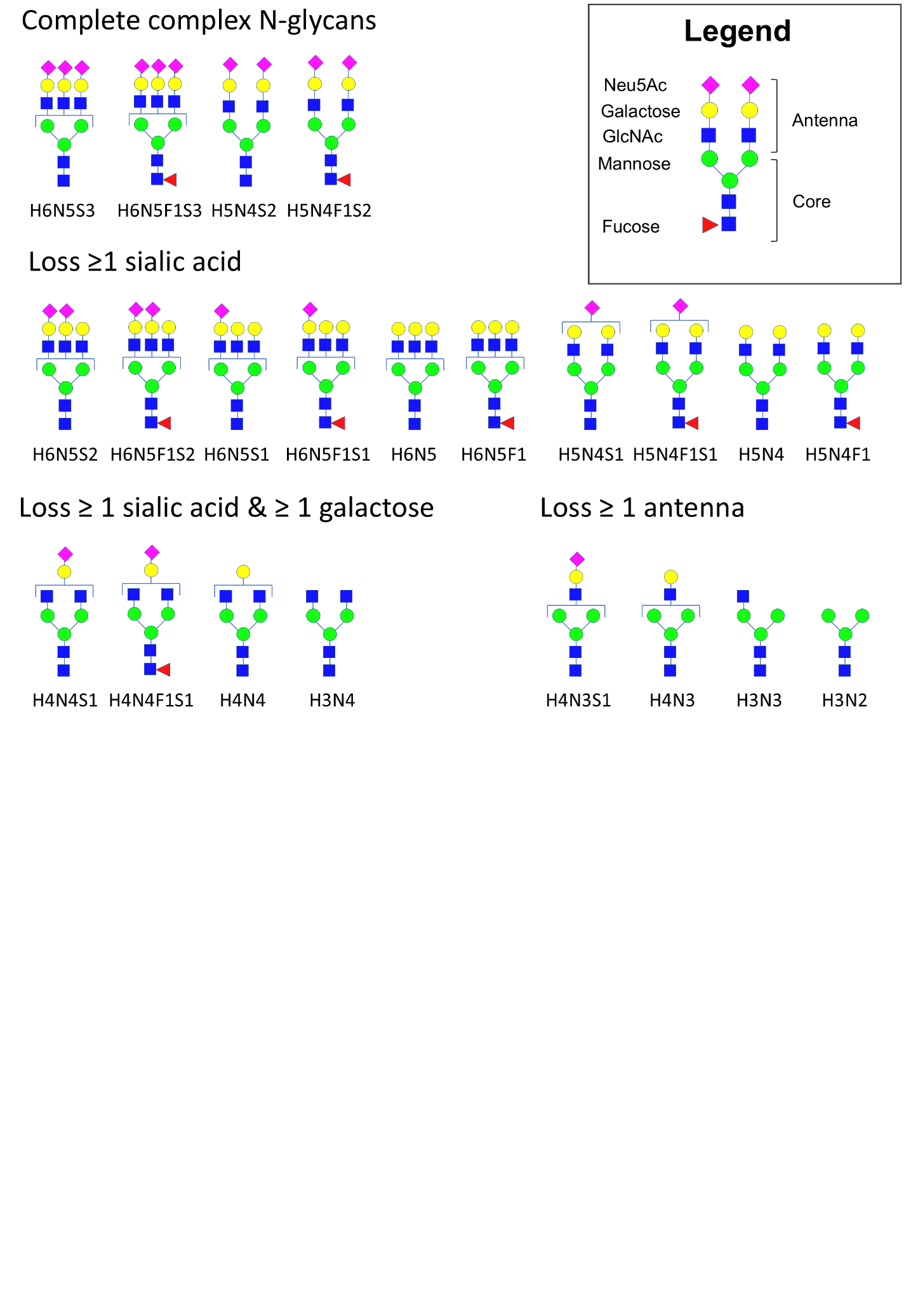
Supplementary Figure 2, glycans included in subgroups.** *Schematic representations of glycans included in each category in this study. The figure legend is shown on the top left: symbols represent sialic acid (Neu5Ac, pink diamond), galactose (yellow circle), N-acetylglucosamine (GlcNAc, blue square), mannose (green circle) and fucose (red triangle).*

**
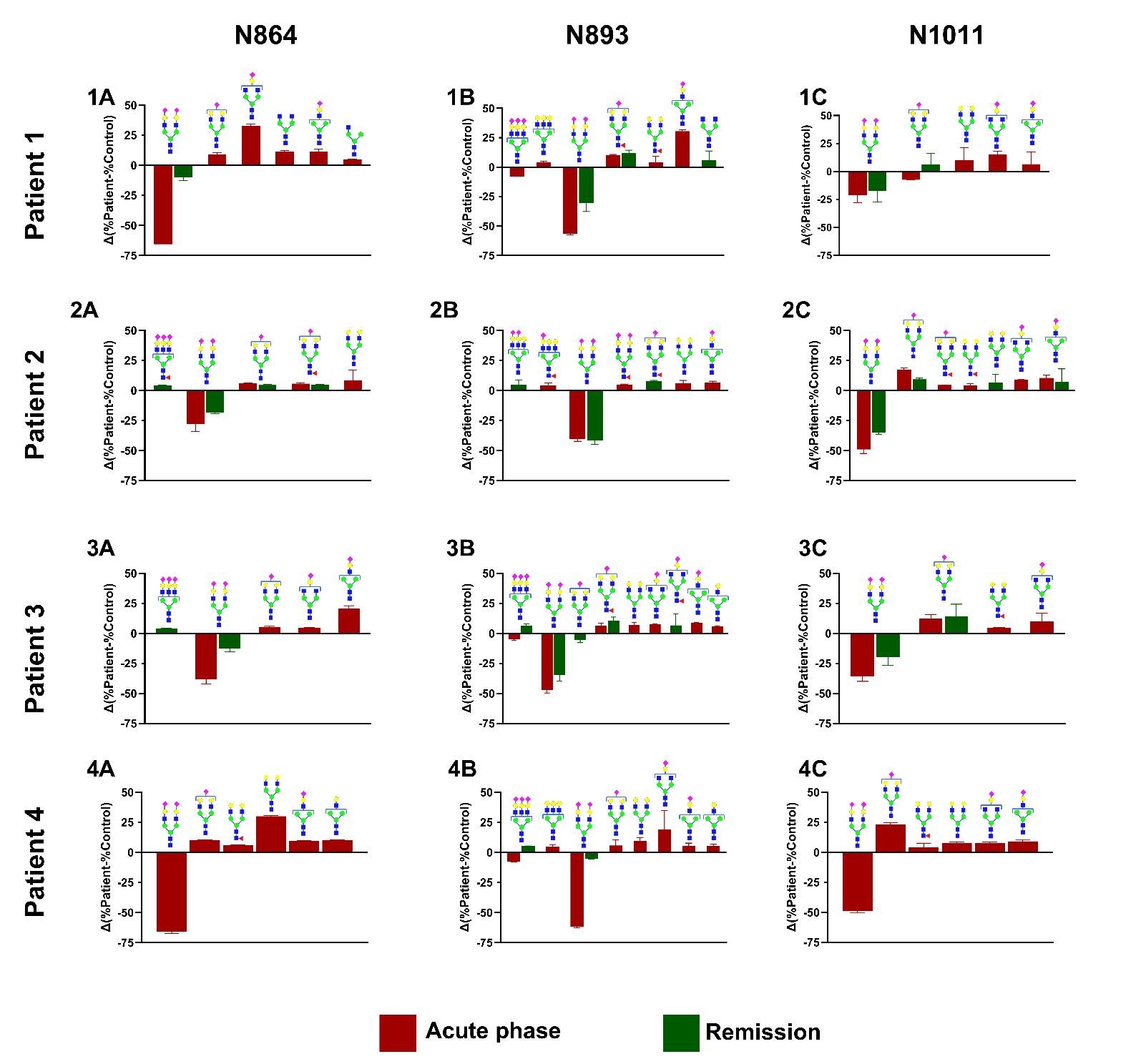
Supplementary Figure 3, glycopeptide profiling.** *Glycosylation status of SP-HUS patient FH. Glycosylation status of three glycopeptides of FH are shown in panel 1A-4A (IPCSQPPQIEHGTNSSR; N864), panel 1B-4B (ISEENETTCYMGK; N893) and panel 1C-4C (MDGASNVTCINSR; N1011). Patients 1-4 were analyzed during the acute phase (red bars) and remission (green bars). Glycosylation changes are expressed as Δ%, where a negative value depicts increase, and negative values a decrease of the abundance of the respective glycoform relative to the controls. Glycoforms with a presence between -4% and 4% are not shown. Values represent the mean of two measurements ± SD.*


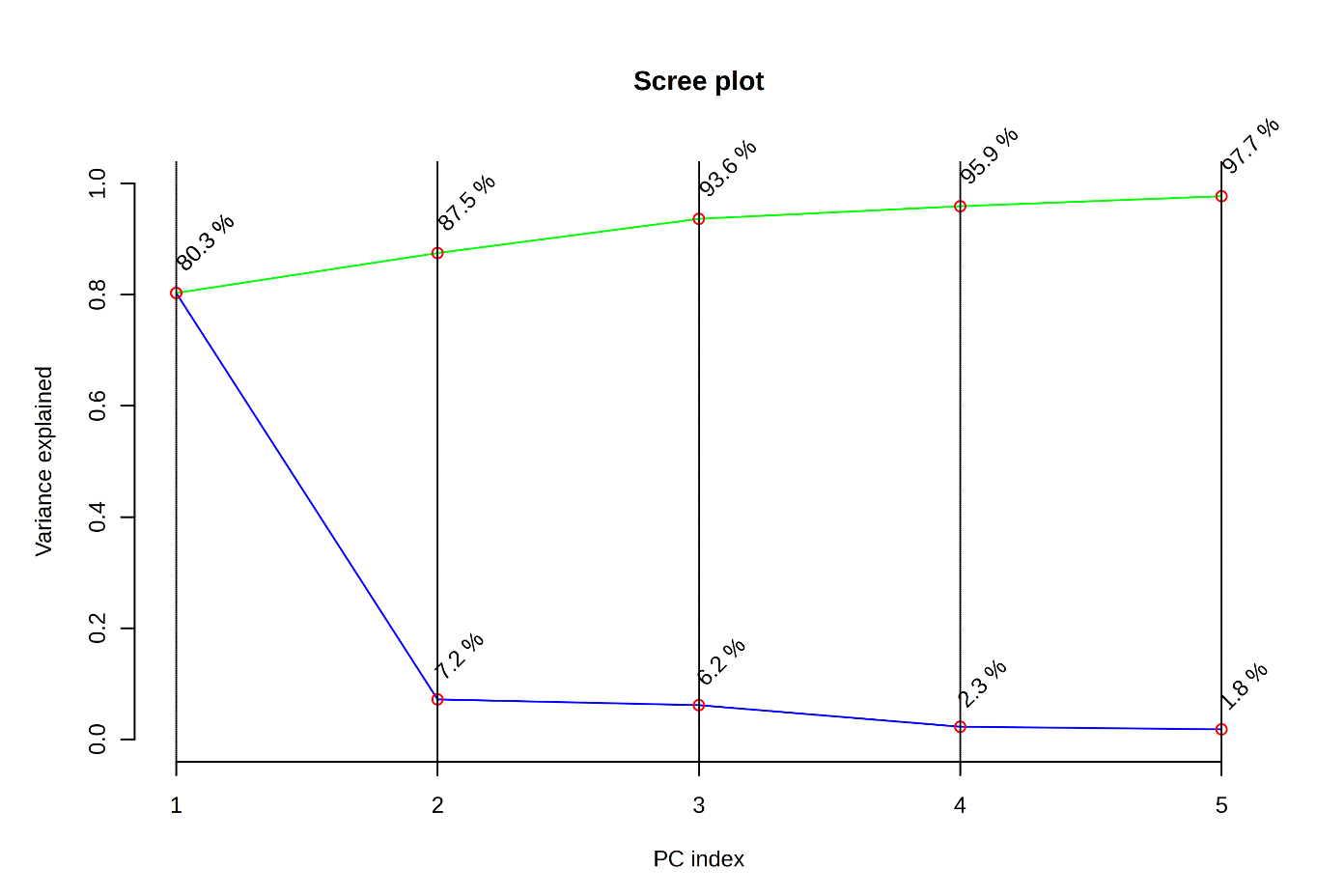
**
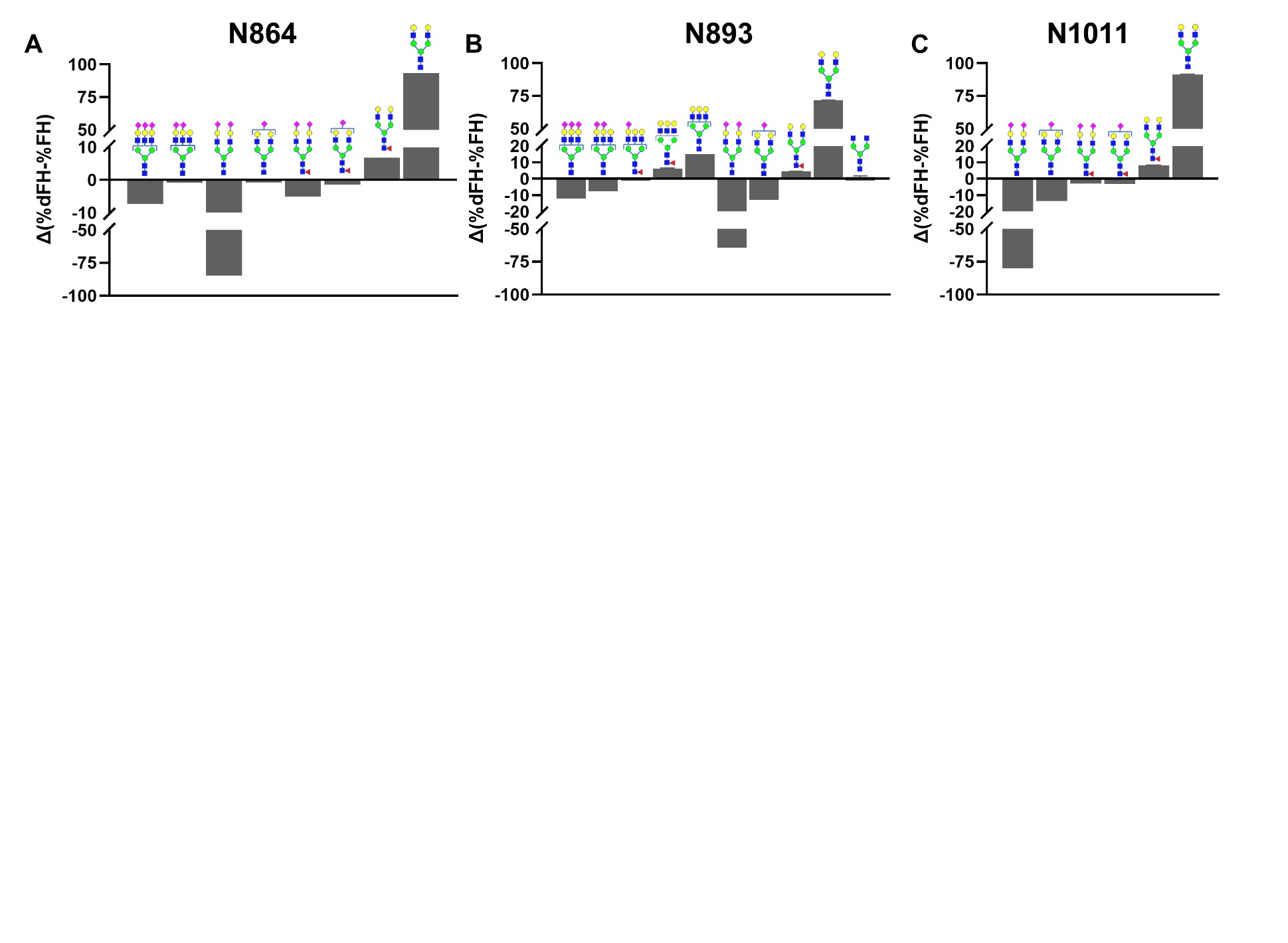
Supplementary Figure 4, loss of sialic acids on Factor H in neuraminidase treated serum.** *Glycosylation status of in vitro desialylated Factor H (dFH) from neuraminidase-treated serum compared to control FH not treated with neuraminidase (FH). Glycosylation status of three glycopeptides of FH are shown: (A): IPCSQPPQIEHGTINSSR, N864; (B): ISEENETTCYMGK, N893; and (C) MDGASNVTCINSR, N1011. Glycosylation changes are expressed as Δ% relative to control FH not treated with neuraminidase. Positive values depict an increase, and negative values depict a decrease of the abundance of the respective glycoform of dFH when compared to the glycoform presence on FH. Values are presented as the mean of two measurements ± SD.*

**Supplementary Figure 5, scree plot of PCA analysis depicted in figure 2.** *Corresponding scree plots from the PCA analysis shown in figure 2D & 2E*

## Supplementary Tables

|  | | P1 | P2 | P3 | P4 |
| --- | --- | --- | --- | --- | --- |
| Sex | | Female | Female | Female | Male |
| Age at onset | | 6 mo | 10 mo | 30 mo | 39 mo |
| Pneumococcal infection | | | | | |
| Kind of infection | | Pneumoniae | Pneumoniae with empyema | Pneumoniae | Pneumoniae with empyema |
| Evidence of invasive *S. pneumoniae* infection (diagnostic test) | | Yes  (urine antigen test) | Yes  (urine antigen test) | - | Yes  (blood culture) |
| Laboratory evidence of HUS | | | | | |
| Hematological TMA | | Yes | Yes | Yes | Yes |
| AKI | | Yes | Yes | Yes | Yes |
| Hospital course | | | | | |
| Hospital stay (days) | | 31 | 23 | 13 | 47 |
| ICU admission | | Yes | Yes | NS | Yes |
| Invasive mechanical ventilation | | Yes | Yes | No | No |
| Extrarenal complications | | No | No | No | No |
| Treatment | | | | | |
| Antibiotics | | Yes | Yes | Yes | Yes |
| Need for dialysis (duration) | | Yes (8 d) | No | Yes (4 d) | Yes (29 d) |
| Antihypertensive drugs | | Yes | Yes | Yes | Yes |
| Number of erythrocytes transfusions | | 7 | 3 | 3 | 6 |
| Eculizumab | | No | Yes (one dose) | No | No |
| Other treatment | |  | VATS | Thoracentesis |  |
| Renal outcome | | | | | |
| Follow-up >6 months after presentation | | No hypertension, no proteinuria, eGFR >90 | No hypertension, no proteinuria, eGFR >90 | No hypertension, no proteinuria, eGFR >90 | No hypertension, no proteinuria, eGFR >90 |
| Exclusion of other causes of HUS | | | | | |
| ADAMTS13 activity | | >10% | >10% | NS | NS |
| STEC | Antibodies against LPS O157, O26, O55, O103 | Negative | NS | NS | Negative |
|  | Fecal PCR/culture | Negative | Negative | Negative | Negative |
| Anti factor H autoantibodies | | NS | Negative | NS | Negative |
| Genetic analysis of complement genes | | No disease-causing genetic variants | No disease-causing genetic variants | NS | No disease-causing genetic variants |

**Supplementary Table 1, clinical parameters of SP-HUS P1-P4.** *Abbreviations: ADAMTS13, a disintegrin and metalloproteinase with a thrombospondin type 1 motif, member 13; AKI, acute kidney injury; d, days; eGFR, estimated glomerular filtration rate; HUS, hemolytic uremic syndrome; ICU, intensive care unit; LPS, lipopolysaccharide; mo, months; NS, not screened; PCR, polymerase chain reaction; STEC, shiga toxin-producing Escherichia coli; TMA, thrombotic micro-angiopathy; VATS, video-assisted thoracic surgery.*

| **Patient** | **Sample** | **Sample information** |
| --- | --- | --- |
| P1 | Acute | Day 3 of hospital admission  Day 1 after TMA presentation |
|  | Remission | 118 days after acute sample |
| P2 | Acute | Day 9 of hospital admission  Day 1-3 after TMA presentation |
|  | Remission | 11 days after acute sample |
| P3 | Acute | Day 3 of hospital admission  Day 1 after TMA presentation |
|  | Remission | 15 days after acute sample |
| P4 | Acute | Day 5 of hospital admission  Day 1 after TMA presentation |
|  | Remission | 135 days after acute sample |

**Supplementary Table 2, sample information of SP-HUS patient samples evaluated during this study.** *Acute samples were drawn 1-3 days after TMA onset, and remission samples were drawn after patients were in hematological remission with normalized platelet counts.*

| **Glycan group** | **Acute phase** | **Remission** | **Healthy controls** |
| --- | --- | --- | --- |
| Complete complex N-glycans | 37.1 (11.3) | 71.3 (11.2) | 86.3 (1.6) |
| Complex N-glycan with loss of ≥ 1 sialic acid | 36.2 (10.1) | 6.0 (13.3) | 12.0 (0.7) |
| Complex N-glycan with loss of ≥1 sialic acid and ≥1 galactose | 11.0 (11.1) | 3.5 (2.0) | 0.2 (0.3) |
| Complex N-glycan with loss of ≥ 1 antenna | 11.7 (3.8) | 1.7 (2.5) | 0.5 (0.9) |

**Supplementary Table 3, presence of glycan groups in SP-HUS patients P1-P4 during the acute phase and remission and in three healthy controls.** *Schematic representations of glycans included in each group can be found in supplementary figure 1. Data is depicted as mean (±SD).*

| **Beads** | **Sample** | **Fraction** | **FH concentration (µg/mL)** |
| --- | --- | --- | --- |
| anti-FH | NHS | IP eluate | 10,1 |
|  |  | Unbound fraction | 24,7 |
|  | ΔFH | IP eluate | 0 |
|  |  | Unbound fraction | 0 |
| hu-IgG | NHS | IP eluate | 0 |
|  |  | Unbound fraction | 46,7 |

**Supplementary Table 4, FH levels determined in the immunoprecipitated (IP) and unbound fractions for controls of the IP using ELISA.** *IP fractions were prepared by incubating anti-FH coupled beads (anti-FH) or beads coupled to human IgGs (hu-IgG) three times with 12.5% control serum (normal human serum, NHS) or serum depleted from FH (ΔFH) in RIPA buffer followed by elution. The unbound fraction contains all serum material that was not bound by the beads. Values represents the mean of one ELISA, all samples were measured in two dilutions in duplicate.*

| **Sample** | **Fraction** | **Neuraminidase activity (pmol/hour/mL)** |
| --- | --- | --- |
| NHS treated with *C. welchii* neuraminidase | FH IP eluate | 196 (495) |
|  | Unbound fraction | 67033 (2479) |
| NHS treated without *C. welchii* neuraminidase | FH IP eluate | -78 (286) |
|  | Unbound fraction | 391 (437) |

**Supplementary Table 5,** **neuraminidase activity assay performed on the immunoprecipitated (IP) fractions and the unbound fractions using anti-FH beads.** *IP fractions were prepared by incubating anti-FH beads three times with 12.5% serum in RIPA buffer followed with elution. The unbound fraction contains all serum material that was not bound by the anti-FH beads. Values represent two measurements (± SD).*
